# Cesarean section rates in Ecuador: a 13-year comparative analysis between public and private health systems

**DOI:** 10.26633/RPSP.2017.15

**Published:** 2017-02-08

**Authors:** Esteban Ortiz-Prado, Tamara Acosta Castillo, Mauricio Olmedo-López, Luciana Armijos, Darío Ramírez, Ana Lucia Iturralde

**Affiliations:** 1 Faculty of Medicine Universidad de las Américas Quito Ecuador; 2 Faculty of Medicine Universidad Central del Ecuador Quito Ecuador; 3 Ministry of Public Health Quito Ecuador; 4 Enfarma EP Quito Ecuador; 5 Faculty of Medicine Universidad Tecnológica Equinoccial Quito Ecuador

**Keywords:** Cesarean section, parturition, delivery of health care, Ecuador.

## Abstract

**Objective.:**

To demonstrate the prevalence of cesearean sections (C-sections) in Ecuador and their distribution between private and public health centers.

**Methods.:**

An observational population-based study was conducted of patients discharged from public and private hospitals in Ecuador after a C-section or vaginal delivery. Data were collected by the Ecuadorian National Institute of Statistics and Census (INEC) between 2001 and 2013.

**Results.:**

The overall national C-section rate in the private health care system is double the rate in the public health care system. Over the 13 years of the study, C-sections accounted for 57.5% of births in the private sector, while the public sector proportion did not exceed 22.3%. Countrywide, less than 36% of C-sections were found to be clinically justified by parallel analysis of absolute or relative indications. Acute fetal distress (AFD) was more frequently reported in private centers compared to public ones (446 per 10 000 live births versus 274 per 10 000). Since 2001, the number of births by cesarean section increased by more than 50% (R^2^ = 0.7306, P < 0.05), with an annual growth rate of 4.03%. In Guayaquil, the largest city in Ecuador, up to 74% of live births occurred by C-section.

**Conclusion.:**

National data show that C-sections are performed more frequently in Ecuador than the rate recommended by the World Health Organization, especially in the private health care system. Private centers also report higher rates of AFD, which implies that this diagnosis is either overused in private centers or underrecognized in public centers. Although several factors might be influencing these trends, no data are available to determine the relative importance of economics, practicality, and medical or personal concerns of mothers and physicians in deciding which method of delivery should be used.

## RESUMEN

**Objetivo.:**

Demostrar la prevalencia de las cesáreas en Ecuador y su distribución entre centros privados y públicos de salud.

**Métodos.:**

Se realizó un estudio de observación basado en la población de pacientes dadas de alta de hospitales públicos y privados en Ecuador después de una cesárea o un parto vaginal. Los datos fueron recopilados por el Instituto Nacional de Estadísticas y Censo (INEC) de Ecuador entre el 2001 y el 2013.

**Resultados.:**

La tasa nacional de cesáreas en el sistema privado de atención de salud es el doble de la tasa que se observa en el sistema público de salud. Durante los 13 años que duró el estudio, las cesáreas representaron 57,5% de los nacimientos en el sector privado, mientras que la proporción en el sector público no superó 22,3%. A nivel de todo el país, se observó que menos de 36% de las cesáreas estaban clínicamente justificadas con un análisis paralelo de indicaciones absolutas o relativas. Se notificaron casos de sufrimiento fetal agudo con mayor frecuencia en los centros privados comparados con los públicos (446 por 10 000 nacidos vivos frente a 274 por 10 000 n.v.). Desde el 2001, el número de nacimientos por cesárea aumentó más de 50% (R^2^ = 0,7306, P < 0,05), con una tasa de crecimiento anual de 4,03%. En Guayaquil, la ciudad más grande de Ecuador, hasta 74% de los nacidos vivos nacieron por cesárea.

**Conclusiones.:**

Los datos nacionales muestran que las cesáreas se realizan en Ecuador con una frecuencia mayor a la tasa recomendada por la Organización Mundial de la Salud, especialmente en el sistema privado de atención de salud. Los centros privados también notifican tasas más altas de sufrimiento fetal agudo, lo que implica que este diagnóstico se utiliza excesivamente en los centros privados o no se lo reconoce sufi- cientemente en los centros públicos. Aunque varios factores podrían estar influyendo sobre estas tendencias, no se tienen datos para determinar la importancia relativa de los factores económicos, la practicidad y las inquietudes médicas o personales de las madres y los médicos al decidir el método de parto.

The global population was estimated to be more than 7 billion in 2014, and the United Nations Children’s Fund (UNICEF) estimates that on average 350 000 children are born each day worldwide (1–2). Birth rates vary from country to country. The method of delivery is highly influenced by cultural traditions and health care access (3–7). In the last 50 years, due to the significant improvement in health care systems, progressively greater numbers of newborns have been delivered within medically controlled settings such as hospitals and small clinics. Even though these factors have contributed to a reduction in prenatal morbidity and mortality rates worldwide, some reports indicate they have also led to overutilization of unjustified procedures and, consequently, increased health care expenditures (8–11). Obstetrician and maternity-related medical services have increased in the last decades, including the number of elective cesareans sections (C-sections) (6, 12). Although C-sections have been shown to be effective in reducing maternal and neonatal mortality in some clinical circumstances, the World Health Organization (WHO) recommends that no more than 15% of newborns should be delivered by this method, while some newer reports suggest that 19% is the level associated with better maternal and fetal outcomes (13–18).

Despite the WHO recommendation and the recent findings of Molina et al. (18), several studies worldwide demonstrate that the proportion of C-sections has increased considerably in the last 50 years, from less than 5% of deliveries to as high as 80% in some countries (6, 16, 19, 20). China reports that more than 50% of their 16 million births every year are delivered by C-section (19, 21). In Brazil this proportion is even higher, reaching more than 80% of total births, especially among those attended in private medical centers (22–24). Throughout Latin America, an estimated 38% of the 10 million births every year occur by C-section, but analyses of the use of C-section versus vaginal delivery are scarce in that region (17, 24, 25).

Although the most dramatic increases have been reported from developing countries, important changes have also been described in countries such as the United States of America, Australia, and Italy (25). In the United States, for instance, the number of reported C-sections increased by more than 48% from 1996 to 2011 (26). Such reports suggest that C-section is one of the most common surgical procedures in for-profit health centers, generating higher health care expenditures (22, 23).

The increase in the number of C-sections is not always clinically justified by prenatal complications (27, 28). Rather, it can be related to women’s anxiety and fear of pain and postpartum complications, as well as sociodemographic factors such as misuse of private insurance or the economic interests of hospitals or physicians (23–25, 27).

In Ecuador, the only data available on the number of vaginal deliveries versus C-sections is a 2014 report from the Ministry of Public Health indicating that more than 35% of births were by C-section (29); no other study was found. Data from the National Institute of Statistics and Census (INEC) show that C-section was one of the leading causes of hospital discharges in 2013, accounting for 8.8% compared to 14.5% for single spontaneous delivery (29, 30). These general nationwide data do not shed light on the relationship between type of birth and prenatal complications or maternal indications for C-sections in neither the public or the private health care system.

The objective of this analysis is to demonstrate the prevalence, geographic distribution, and trends over time of Csections performed in private versus public health centers and to investigate their clinical justification.

## METHODS

This observational population-based study describes the available data related to C-sections and vaginal deliveries in Ecuador over a 13-year period. Data sources included annual hospital discharges and maternal and neonatal morbidity and mortality information available through INEC from 2001 to 2013. Births were categorized according to the 10th edition of the International Classification of Diseases (ICD-10) as vaginal delivery or cesarean section.

All births reported to local health authorities from 2001 to 2013 were included in the analysis and were disaggregated by region, province, city, and type of health care facility (public or private). Reports from the social security system were added to the public health care data, as was information from military, naval, and police hospitals. Information from the insular region (Galápagos Islands) was incomplete but was included where available. The socioeconomic characteristics included in the analysis were age, province of residency, type of establishment where delivery took place, and number of days hospitalized.

A descriptive analysis, including measures of central tendency and dispersion, was performed. Annual growth rate calculation and univariate analysis were done using SPSS version 20 and Microsoft Office for Windows 2013. Maps were made using QGIS version 2.6.1 and the data were stored in a Microsoft Office Excel file. Reference citations and retrieval were managed using Zotero version 4.0.11.

## RESULTS

**FIGURE 1.**
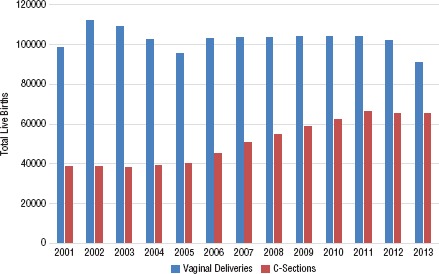
Number of live births by vaginal delivery and by cesarean section (C-section), Ecuador, 2001–2013

***Source***: INEC data.

In Ecuador in the 13-year period from 2001 to 2013, a total of 1 796 826 live births were officially reported. During this period the number of live births by C-section increased by more than 50% (R^2^ = 0.7306, *P* < 0.05). The annual growth rate of C-section births in Ecuador was 4.03% (Figure 1). The overall national Csection rate in the private health care system was double the rate in the public health care system (575 per 1000 versus 223 per 1000 live births).

### Maternal age

The data show that 60% of live births took place among mothers between 20 to 34 years old, while more than 22% of live births were to teenage mothers from 10 to 19 years old.

An inverse relationship (*P* < 0.05, R^2^ = 0.8785) was observed between the percentage of live births by vaginal delivery and maternal age. Older mothers have higher rates of C-section than younger mothers.

### Geographic distribution

An analysis by geographic area found that in the coastal region more than 44% of the total live births were by C-section. In the highland provinces, the proportion was under 18%, and in the Amazonian provinces it was below 13%. The annual growth rate of C-sections in the coastal region corresponded to 11.3%, while in the highlands it was 4.6%.

### Urban versus rural

The data indicate that most C-sections are performed in large urban areas. A high percentage of births by C-section are reported in the largest cities (Table 1). In 2011, Guayaquil was the city with a higher percentage of C-sections (74%), with an annual net increase of over 10% during the study period. On the other hand, in Quito and Cuenca, the average percentage of C-sections decreased slightly after 2005.

### Public versus private

**FIGURE 2.**
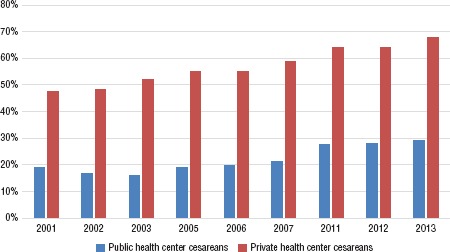
Percentage of live births by cesarean section in public and private health centers, Ecuador, selected years between 2001 and 2013

***Source: INEC data.***

From 2001 to 2005, the number of C-sections in Ecuador stayed almost constant. However, from 2005 to 2011, there was a noticeable linear increase (R^2^ = 0.992; *P* < 0.001), with the number of C-section deliveries rising by an average of 4322 per year; in 2012–2013 C-sections showed a small decrease in absolute numbers but not percentage (Figure 2).

In absolute numbers, the private health care system reported fewer live births than the public health care system (412 316 versus 1 384 510). However, within the private system the rate of C- sections per 1000 live births (575 per 1000) was double the rate in the not-for- profit public health system (223 per 1000). Over the 13-year study period, 57.5% of births in the private sector were by C-section, while in the public sector the proportion did not exceed 22.3%.

**TABLE 1.** Consolidated rate of cesarean sections (C-sections) between 2001 and 2013 in three major cities in Ecuador

City	Total no. of live births^a^	No. of C-sections^b^	C-section rate per 1000 newborns^c^
Guayaquil	178 113	98 042	551
Quito	180 694	35 515	197
Cuenca	54 597	13 587	249

^a^ Data from Instituto Nacional de Estadística y Censos (INEC), 2013. ^b^ Mean of the yearly total from 2001 to 2013. ^c^ Number of C-sections divided by the total number of live births from 2001 to 2013.

**FIGURE 3.**
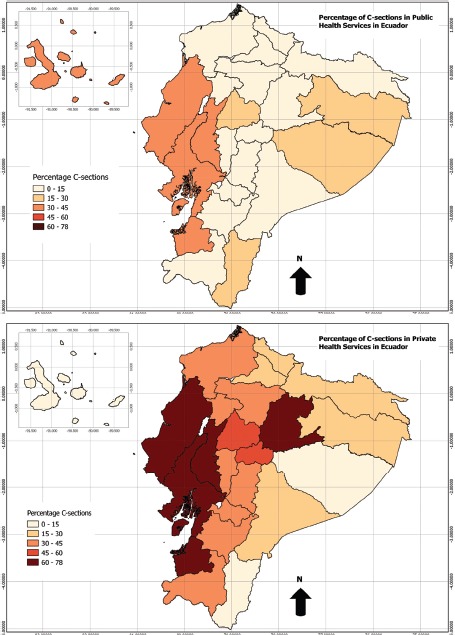
Proportion of live births delivered by cesarean section (C-section) in the public (top map) and private (bottom map) health services, by province, Ecuador, 2001–2013

***Source: INEC data.***

By region, C-sections represented 61% of total live births reported in the private sector in the coastal area, 36% in the highlands, and 28% in the Amazon basin. Rates of C-section births in the private sector were alarmingly high in some provinces, including El Oro, Los Rios, and Guayas. In Manabí, one of the most populated provinces on the coast, C-sections accounted for 78% of total live births (Figure 3). Among the highland provinces, Cotopaxi and Tungurahua had the highest percentage of C-sections in the private health care sector, with 51% and 55% respectively.

### Clinical justification for C-sections

Analysis of data from INEC showed that 51% of C-section births in the public sector could be justified by parallel reports of prenatal maternal or fetal complications, including prenatal hypoxia, multiple pregnancy, or labor dystocia and other absolute or relative indications for C-section. However, in the private sector only 22% appeared to have such justification.

**FIGURE 4.**
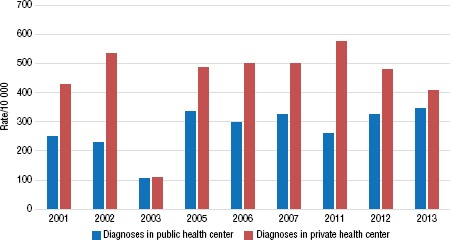
Rate (per 10 000 live births) of acute fetal distress diagnosed in public and private health centers, Ecuador, selected years between 2001 and 2013

***Source: INEC data.***

An interesting finding was the frequency of occurrence and diagnosis of acute fetal distress (AFD). The overall data from 2001 to 2013 demonstrate that AFD was more frequently reported in private health centers than in the public system (446 per 10 000 births versus 274 per 10 000) (Figure 4).

## DISCUSSION

The results confirm that the proportion of births by C-section in Ecuador increased by more than 50% between 2001 and 2013. The procedure was most common among mothers in urban areas in coastal provinces, and the overall national C-section rate in the private health care system was double the rate in the public health care system (575 per 1000 versus 223 per 1000 live births).

The progressive increase in the number of births by C-section in Ecuador reached an annual growth rate of 4.03% during the study period. These data support previous international studies that ranked Ecuador in second place among Latin American countries with the highest C-section rates (31).

In three of the four regions of the country, the average percentage of C-section deliveries exceeded the percentage of vaginal deliveries. The Amazonian region was the only area where the overall C-section rate (private and public systems) did not exceed the 15% recommended by WHO (15–17).

The provinces located on the coast had higher rates of C-section than those located in the highlands and the Amazon basin. These findings could be explained by the larger indigenous population in the highlands and Amazonian provinces, a demographic characteristic usually related to higher lactation rates (32–34).

In Ecuador, C-section rates were significantly higher in urban areas. These results were similar to reports from China, Brazil, Singapore, and Hong Kong, where urban areas account for the majority of C-sections (4, 22, 27). The largest urban areas and cities in Ecuador have the highest rates of C-section. For instance, in Guayaquil, the largest city in Ecuador, an alarming 74% of live births occurred by C-section within the private health care system, the rate growing more than 10% annually.

Worldwide, the increase in C-sections has been attributed to perinatal factors such as continuous fetal monitoring, previous C-section, increasing maternal age at delivery, prenatal anxiety, fear of pain, and cultural beliefs (16, 17, 19, 35, 36). In Ecuador, our results demonstrated that 51% of C-sections reported by the public health system and only 22% within the private for-profit health system were justified by parallel reports of maternal or fetal complications. The extent to which personal preference and prior delivery by C-section influenced these rates could not be determined from the INEC data or the ICD-10 coding.

Countrywide, fewer births per year take place in the private sector, but the overall rate of C-sections per 1000 births is significantly higher than in the free public system. This difference between the public and private health care systems merits particular attention since access to private for-profit centers was found to be an important determinant for having a C-section procedure (37).

The reasons why mothers who can afford private health care have a higher proportion of C-sections are not fully understood. However, studies from China and Brazil have implicated financial incentives that promote C-sections, such as insurance coverage in wealthier populations, higher physician remuneration, and higher hospital profits for a C-section than for a normal vaginal delivery (4, 17, 28, 38, 39).

Interestingly, the private health care system reported on average 43.4% more C-sections that were clinically justified by the occurrence of acute fetal distress (AFD) or prenatal hypoxia than the public health system (446 per 10 000 versus 274 per 10 000 live births). Although it might be argued that the higher rate of prenatal hypoxia within the private health system resulted from superior prenatal monitoring, deliberate misdiagnosis can not be ruled out in all cases as an excuse to perform unnecessary C-sections.

According to the 2014 public medical fee for service table (40), an uncomplicated C-section should cost US$ 892, while a normal uncomplicated vaginal delivery in the public health system can cost up to US$ 658. Using our data, we hypothesize that at least 130 000 private-sector and 108 000 public-sector C-sections were performed unnecessarily between 2001 and 2013, representing more than US$ 115 million (private) and US$ 92 million (public) in unnecessary health expenditure in those years.

Although it would be unjustified to draw any conclusions about causality, unpublished data suggest that maternal disempowerment, maternal fear, physician’s convenience and practicality, and economic remuneration might be affecting C-section rates in Ecuador. Possible solutions include higher medical remuneration for vaginal parturition, longer paternal leave when a newborn has been delivered normally, mandatory institutional assistance for those mothers seeking normal vaginal deliveries, promotion of the benefits of humanized delivery, and better definition among specialists of the absolute and relative indications for C-section applicable in Ecuador.

### Study limitations

Use of nationally representative discharge data obtained from hospital discharge inputs relies on accurate coding. Errors of omission and commission may occur. Data regarding repeat or elective C-sections were not available.

### Conclusions

Since 2001, Ecuador has seen an important increase in the rate of C-sections. Geographic and sociodemographic characteristics, as well as provider and patient preferences, have each contributed more to the current high C-section rate than have clinical justifications.

Private urban medical centers on the coast of Ecuador are responsible for the majority of unnecessary C-sections, while public rural medical centers, especially those serving large indigenous populations, maintain lower rates of unnecessary C-sections. Fewer children are delivered within the private health sector, but the rate of C-section is significantly higher than in the public sector, as is the number of children with acute fetal distress.

Most mothers reported that their pregnancies ended in C-section due to the physician’s recommendation, while the proportion of clinically justified C-sections was 22% in private health centers and 51% in public health centers.

### Conflicts of interest.

None.

### Disclaimer.

The authors holds sole responsibility for the views expressed, which may not necessarily reflect the opinion or policy of the *RPSP/PAJPH* or the Pan American Health Organization (PAHO).
